# Soil carbon sequestration potential in global croplands

**DOI:** 10.7717/peerj.13740

**Published:** 2022-07-21

**Authors:** José Padarian, Budiman Minasny, Alex McBratney, Pete Smith

**Affiliations:** 1 Sydney Institute of Agriculture & School of Life and Environmental Sciences, University of Sydney, Sydney, Australia; 2 Institute of Biological & Environmental Sciences, University of Aberdeen, Aberdeen, United Kingdom

**Keywords:** Carbon sequestration, Quantile regression, Digital soil mapping, Neural networks

## Abstract

Improving the amount of organic carbon in soils is an attractive alternative to partially mitigate climate change. However, the amount of carbon that can be potentially added to the soil is still being debated, and there is a lack of information on additional storage potential on global cropland. Soil organic carbon (SOC) sequestration potential is region-specific and conditioned by climate and management but most global estimates use fixed accumulation rates or time frames. In this study, we model SOC storage potential as a function of climate, land cover and soil. We used 83,416 SOC observations from global databases and developed a quantile regression neural network to quantify the SOC variation within soils with similar environmental characteristics. This allows us to identify similar areas that present higher SOC with the difference representing an additional storage potential. We estimated that the topsoils (0–30 cm) of global croplands (1,410 million hectares) hold 83 Pg C. The additional SOC storage potential in the topsoil of global croplands ranges from 29 to 65 Pg C. These values only equate to three to seven years of global emissions, potentially offsetting 35% of agriculture’s 85 Pg historical carbon debt estimate due to conversion from natural ecosystems. As SOC store is temperature-dependent, this potential is likely to reduce by 14% by 2040 due to climate change in a “business as usual” scenario. The results of this article can provide a guide to areas of focus for SOC sequestration, and highlight the environmental cost of agriculture.

## Introduction

Soil is a major influencer of the global carbon and nutrient cycle and it holds more carbon than all terrestrial vegetation combined. However, the use of soils for food production causes 30–60% of this carbon to be lost, causing a decline in soil functionality (Kopittke et al., 2019). The released carbon diffuses into the atmosphere as carbon dioxide, making agriculture an important contributor to the increasing greenhouse gas emissions (IPCC, 2019). The loss of carbon from soils causes a loss of its productivity and associated ecosystem services (Russel, Harrison & Wright, 1929; Mann, 1986; Davidson & Ackerman, 1993).

The carbon debt from agriculture poses the challenge of reversing the process. Sanderman, Hengl & Fiske (2017) estimated 31.2 Pg C had been lost in topsoil (0–30 cm) through 12,000 years of agricultural practices. SOC sequestration is considered as a viable alternative to recoup part of this debt and to partially mitigate climate change by offsetting part of the greenhouse gas emissions derived from anthropogenic activities (Minasny et al., 2017). Researchers maintain that sustainable soil C sequestration practices need to be scaled up globally (Amelung et al., 2020). However, there are still many conflicting results on how much C can soil potentially store, especially in different regions of the world. SOC stores and dynamics are driven by soil, environmental and human factors such as soil type, climate, land use, and land management. However, SOC storage potential and its controlling factors are not well understood at the global scale. Currently, SOC storage potentials are often expressed as constant, per-year values based on management tables and assuming a uniform time to reach a new equilibrium, which is usually the 20 years default value used by IPCC (Fuss et al., 2018). Additionally, since climate exerts an essential control on SOC and global climatic data is readily available, most studies focus on temperature and precipitation to model SOC retention (Jones et al., 2005; Kramer & Chadwick, 2018; Shi et al., 2020). The effect of climate on SOC might not be well represented due to other confounding, unmeasured variables acting as limiting factors (Cade & Noon, 2003). Management practices are in this category, playing an essential role at the local scale, but detailed global information about management practices is not available to date, the reason why it is usually missing in global studies.

**Figure 1:**
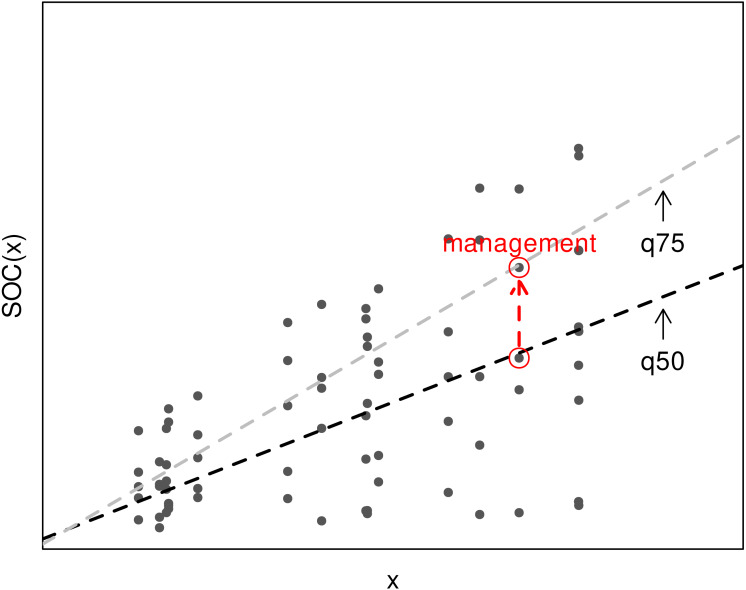
Diagram of a linear quantile regression fitted to the 50th, 75th percentiles to explain SOC content based on a covariate *x*. The points correspond to different sites, where the lower values of SOC are due to unmeasured limiting factors (such as management). A conventional regression model is usually adjusted to the mean (close to q50) but a quantile regression is also capable of capturing the response of high-performing sites (q75). Since our model includes soil, climate and topography, we hypothesise that the difference is mainly due to management practices.

To partially overcome the lack of detailed management data, we create a model that spatially predicts SOC distribution. Given a set of SOC observations from locations with similar soil forming factors (which are accounted by the model), we hypothesise that differences in SOC are mainly driven by management. Different parts of the statistical distribution of SOC should represent locations with different management practices, with “good” practices in the higher quantiles and “bad” practices in the lower quantiles Fig. 1. The model is not aware of the specific management at each location but it is capable of representing different management levels and their impact on SOC given real world data. This can be achieved using the quantile regression framework (Cade & Noon, 2003), the framework we use in this work.

This study aims to provide an estimate of the additional SOC storage potential in global croplands. We focused our analysis on the top 30 cm of soil since it accounts for a large proportion of the SOC stored in soils (Batjes, 1996; Jobbágy & Jackson, 2000), it is considered the depth that can be effectively managed to capture carbon (VandenBygaart et al., 2011), and presents faster turnover time (Shi et al., 2020). Since temperature and precipitation are important controlling factors, we also evaluate how climate change projections might affect this additional storage potential.

## Methods

### Data sources and preparation

The soil information used in this study was derived from several sources, including the World Soil Information Service (WoSIS) (Batjes, Ribeiro & Van Oostrum, 2020), the Chilean Soil Organic Carbon database (CHLSOC) (Pfeiffer et al., 2020), and the data described in Stockmann et al. (2015). Since this is a collation of mostly legacy data, and soil analytical methods change in time, part of the SOC data (Stockmann et al., 2015) were harmonised to a common measuring method using pedotransfer functions (Skjemstad, Spouncer & Beech, 2000). The observations correspond to soil profile data with SOC measurements at varying depths, which were standardised to the 0–30 depth range using the equal-area spline algorithm (Bishop, McBratney & Laslett, 1999). To estimate the soil C stock, we derived a pedotransfer function to predict bulk density using samples from our compiled dataset.

Land cover information was extracted from the MCD12Q1v6 MODIS product, generated by the Land Processes Distributed Active Archive Center, U.S. Department of the Interior and U.S. Geological Survey (DOI: 10.5067/MODIS/MCD12Q1.006), specifically the IGBP classification (Loveland & Belward, 1997) matching the year of each sample.

From the initial 83,416 samples, 5% were held out as a test dataset. The remaining 95% were split into training and validation datasets using the bootstrapping routine (Efron & Tibshirani, 1993) to find the optimal set of hyperparameters of the model. Hyperparameters are the group of parameters of a machine learning algorithm which cannot be learned in the training process (*e.g.*, learning rate, batch size, etc.). Here we report the results of the best performing model after performing a parameter grid search.

The covariates used as predictors included: (a) digital elevation model (GTOPO30 (USGS EROS, 2020)), which is provided at 30 arc-second resolution; and (b) long term mean annual temperature (MAT) and total annual rainfall (TAP) derived from information provided by WorldClim (Hijmans et al., 2005), at 30 arc-second resolution. All data layers were resampled to a 500 m grid and standardised using the mean and standard deviation estimated from the training dataset.

### Quantile CNN model

**Figure 2:**
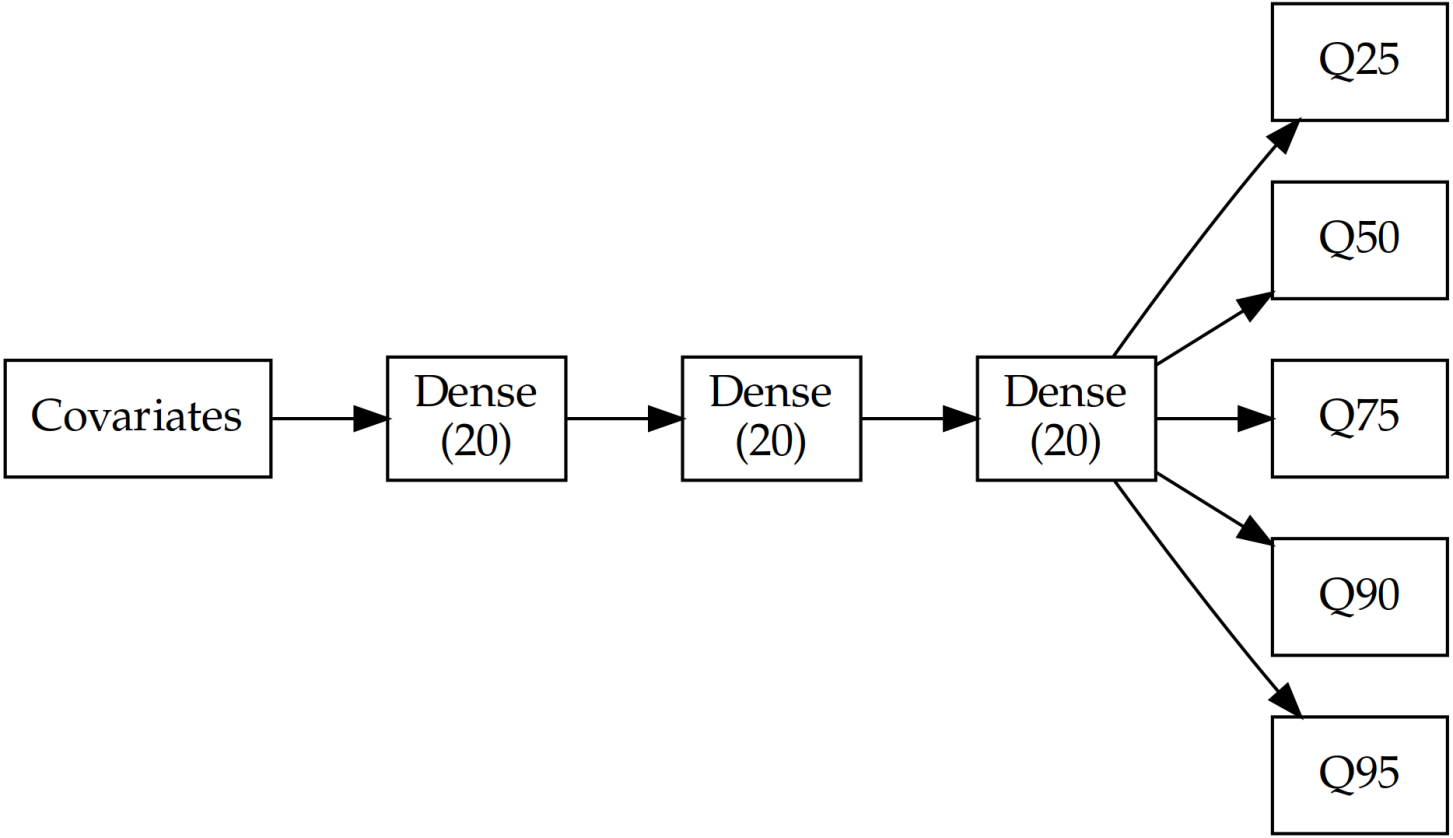
Architecture of the multi-task network. Each branch corresponds to one of the predicted percentiles (25, 50, 75, 90 and 95th).

To model SOC distribution in space, we used a fully-connected, multi-task neural network with three hidden layers of 20 units each and ReLu activation functions. Since we were interested in predicting multiple percentiles of the SOC distribution, the head of the network consisted of five branches of a single unit with linear activation, which corresponds to the 25th, 50th, 75th, 90th and 95th percentiles (Fig. 2). Multi-task neural networks (*i.e.,* that predict multiple targets simultaneously) have shown excellent predictive capability compared with predicting a single target in digital soil mapping and we refer the reader to Padarian, Minasny & McBratney (2019) for a detailed introduction. Each branch can be considered as an independent model (in reality, they share the initial layers) where the training process tries to minimise the loss (Eq. (1)). In a normal regression, the loss is usually the deviation from the mean (using mean squared error) but in this case, it is the distance to each percentile.

The results reported here are based on the predictions at 50th, 75th and 90th percentiles and the 25th and 95th percentiles where included for regularisation purposes (Ruder, 2017). The model was trained using the best set of hyperparameters obtained from a grid search, namely 100 epochs, a batch size of 32 samples and a learning rate of 0.001. For each percentile (branch of the multi-task neural network), the loss is estimated by:

(1) \begin{eqnarray*} \frac{1}{n} \sum _{i=1}^{n}max \left\{ \tau ({y}_{i}-\hat {{y}_{i}}),(\tau -1)({y}_{i}-\hat {{y}_{i}}) \right\} \end{eqnarray*}

as per Koenker & Hallock (2001), where *τ* is the corresponding percentile and *n* is the number of training samples. The final, total loss corresponds to the sum of the five individual losses. We trained different models for croplands, natural/pasture and forest land covers although here we mostly focus on global croplands. Since most global SOC models use a central estimate, such as the mean or median, here we assume the median (50th percentile) as the current state of SOC in the world. Higher quantiles represent situations where better management practices are in place (Fig. 1). These are percentiles are defined by real-world observations with similar climate, soil, topography, and land cover where higher SOC content values can be observed. To avoid considering very extreme cases and ensure that the target SOC content was reached in an important proportion of the locations, we used a regression to the 90th percentile as a technical maximum. Acknowledging that increasing SOC content is a challenging task and this technical maximum might not be always achievable, we considered a regression to the 75th percentile as an intermediate, a technically achievable storage goal.

The model accuracy was evaluated on the test dataset by estimating the root mean squared error (RMSE; Eq. (2))

(2) \begin{eqnarray*}RMSE=\sqrt{ \frac{1}{n} \sum _{i=1}^{n}{ \left( {y}_{i}-\hat {{y}_{i}} \right) }^{2}}\end{eqnarray*}

where $\hat {{y}_{i}}$ corresponds to the prediction of the 50th percentile for the *i*th sample, *y*_*i*_ is the observed *i*th value, and *n* is the total number of observations.

### Model interpretation

To understand how the different covariates control SOC distribution and to corroborate that our model is capturing sound relationships, we used an approximation of Shapley values (Lundberg & Lee, 2017) (SHAP) to estimate the contribution of each covariate to the model predictions. In brief, to estimate the marginal contribution of a covariate, ideally we would like to train models with an without the covariate and evaluate their difference. To correctly estimate the contribution, this process should be done for all the combinations of covariates to consider their interactions. Since this becomes computationally prohibitive due to the exponential complexity of the problem, Lundberg & Lee (2017) developed the SHAP method as an approximation. This method has been recently introduced in soil sciences and has been applied to large extent digital soil mapping showing potential to interpret complex models (Padarian, McBratney & Minasny, 2020), or interpreting soil spectral models (Haghi, Pérez-Fernández & Robertson, 2021).

### Historical carbon debt

While this work focuses on croplands, the model was trained considering croplands, pastures/natural and forest land covers. This allowed us to simulate situations where current croplands are replaced by pastures/natural and forest, but still accounting for local conditions. Without considering the high complexity of land use change and its feedback (Ojima, Galvin & Turner, 1994), land cover substitution was used as a simple proxy to estimate the historical debt due to agriculture. Since our model is capable of predicting different percentiles, we substituted current croplands with (a) the median (50th percentile) of pasture/natural land cover and (b) the 90th percentile of forest land cover to get a range of possible scenarios. As the range was quite wide, the mean was assumed as the historical debt.

### Future climate projections

To estimate the carbon stocks and additional storage capacity under future climate projections, we ran the model using downscaled temperature and precipitation estimates of nine General Circulation Models (BCC-CSM2-MR, CNRM-CM6-1, CNRM-ESM2-1, CanESM5, GFDL-ESM4, IPSL-CM6A-LR, MIROC-ES2L, MIROC6, and MRI-ESM2-0) for the moderately pessimistic SSP3−7.0 scenario, which considers a world that does not enact climate policies. We present the mean estimate of the aforementioned models for the 2021–2040 period. All the estimates were retrieved from the WorldClim 2.1 database (Fick & Hijmans, 2017).

## Results and discussion

### SOC and controlling factors

In terms of accuracy, the model evaluated on the test dataset (5% of the available data) showed a good performance, with a root mean squared error of 3.14% estimated as the deviation from the median (50th percentile). This value is lower than the error reported by the latest release of SoilGrids which obtained an error to the median of 3.97% using a quantile random forest regression (Poggio et al., 2021).

**Figure 3:**
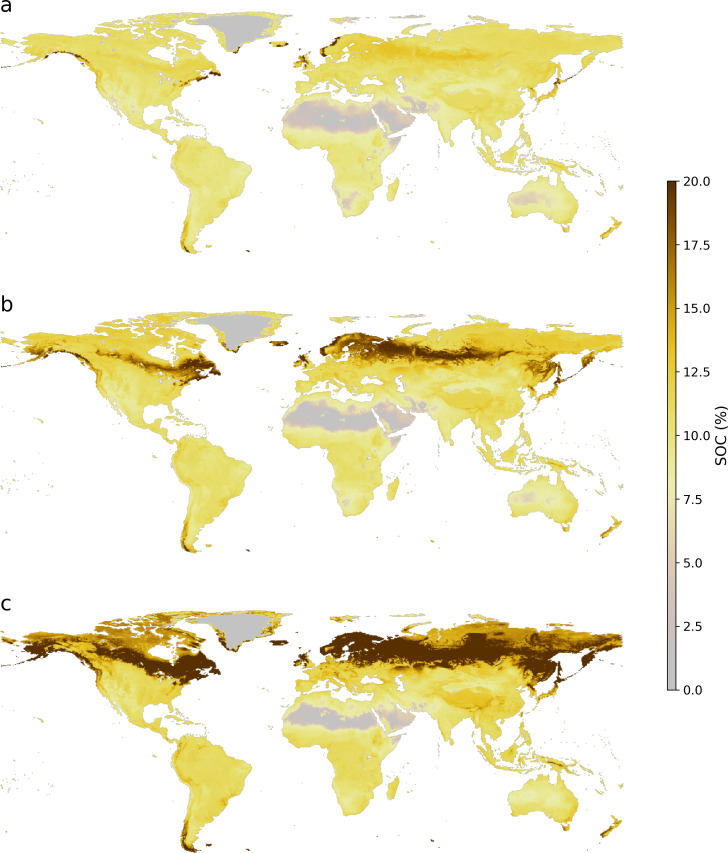
Global maps of topsoil organic carbon predictions under (A) current condition, (B) 75th and (C) 90th percentile.

First, we considered croplands and pastures/natural land covers, and our model showed an upward trend from the current state to the achievable potential and technical maximum, with median SOC contents around 1.47% representing the current state, and 2.06 and 2.81% for the 75th and 90th percentile in croplands. The 75th and 90th percentile in pastures were higher with estimates of 2.43 and 3.43%, respectively. To illustrate the spatial variability of the additional storage potential, we generated global maps of the SOC median, 75th and 90th percentile (Fig. 3). The maps show the expected global SOC trend, with low SOC contents in areas such as the Sahara, Arabian, Gobi, Australian and Atacama deserts, and high SOC contents in the humid tropics and towards the poles. The maps also show the increasing concentrations of SOC in the current, 75th and 90th percentiles (Figs. 3A, 3B and 3C, respectively).

**Figure 4:**
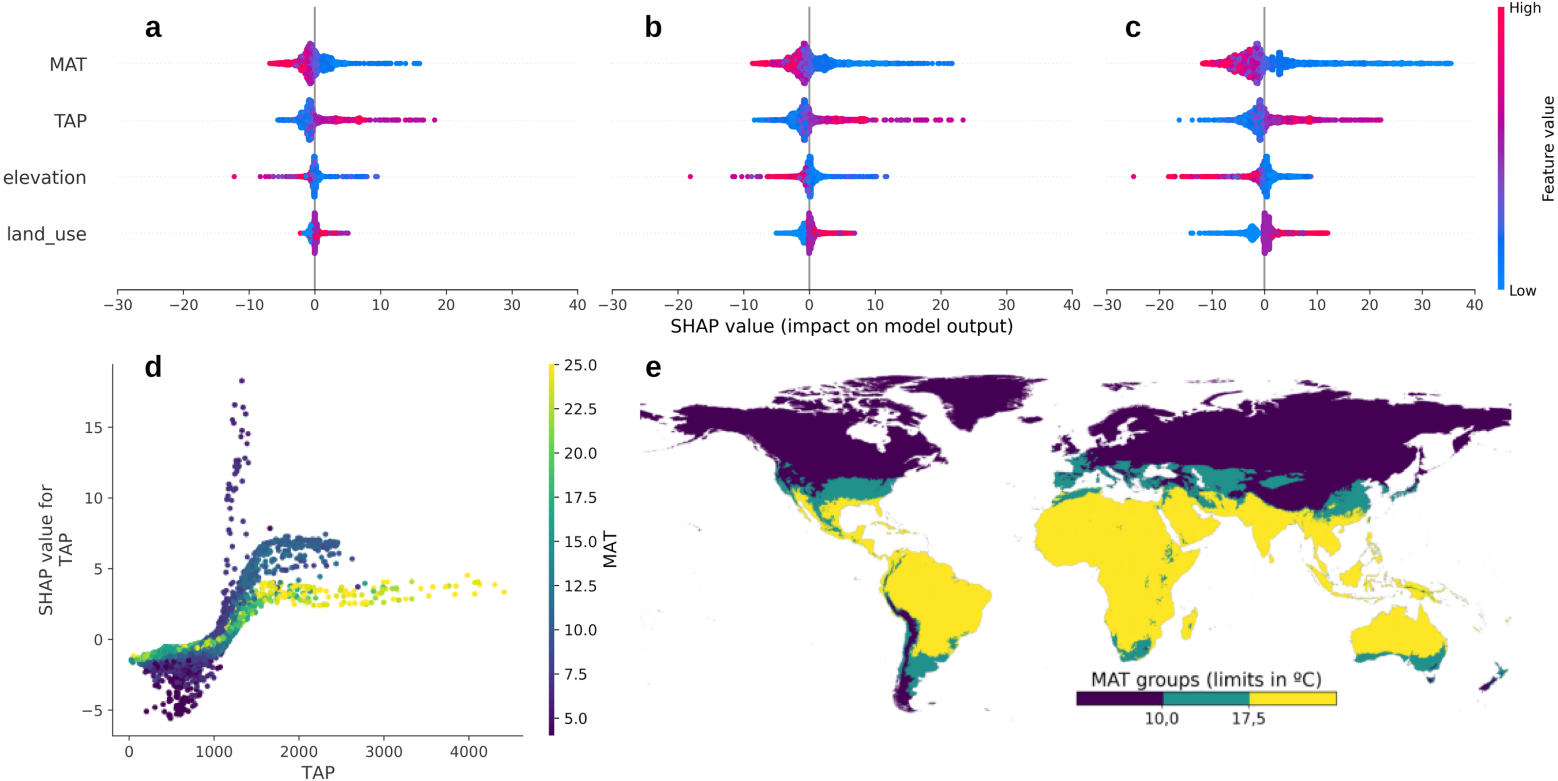
Contribution of each covariate to the final map model predictions and important interactions (SHAP values). Contributions to the 50th (A), 75th (B) and 90th (C) percentiles. The land cover covariate consists of three classes, namely croplands, pastures, and forest (red, purple and blue, respectively). D–E Soil organic carbon dependency on temperature and precipitation. (D) Global distribution of the three identified temperature groups and (E) precipitation dependency (SHAP values).

The SHAP values corroborated that the model captured sensible relationships between the environmental covariates and SOC distribution (Figs. 4A–4D). Here, we describe the SOC dependence on environmental factors, but there are also intrinsic edaphic factors that control soil carbon storage. Soil clay content has been recognised as a key factor in SOC stabilisation (Oades, 1988; Schimel et al., 1994) and, ideally, our model should include this significant relationship. We used global soil texture information (Poggio et al., 2021), however, the resulting model did not show the expected spatial global patterns. This is probably due to the current global texture maps do not capture enough local variation. In consequence, we excluded clay content from our model but we stress the importance of it in local models when good covariates are available.

For all the SOC percentiles, climatic variables had the greatest influence, closely followed by elevation and land cover. All the contributions increased at higher percentiles as a consequence of the higher predicted SOC values. To normalise these values, we calculated the percentage change from the 50th percentile which revealed a greater influence of land cover (64.16 and 257.95% to the 75th and 90th percentiles) when compared to other environmental factors (33.09 and 107.24% for MAT; 33.07 and 60.62% for TAP; 37.29 and 54.92% for elevation). The results clearly show the influence of land cover on SOC stocks. At the median (Fig. 4A), the model assigned a modest negative contribution to land cover. As we move towards higher percentiles, the negative effect of croplands (blue dots in the land cover row) increases substantially, clearly differentiating itself from the other two classes (pastures and forest). The contribution of MAT also increased considerably, particularly for the observations with low temperatures, due to the decrease in carbon turnover (Carvalhais et al., 2014).

The dependence of SOC on temperature and precipitation at the global scale has been thoroughly described in the literature (*e.g.*, Davidson & Janssens (2006)). Our model captured this dependency, showing a clear interaction between both factors. In Figs. 4D–4E, using SHAP values, we can distinguish three groups of SOC responses. First, for temperatures above 17.5 °C, the TAP dependency follows a logistic curve with a relatively low plateau due to the high soil respiration rates and SOC turnover, corresponding mainly to tropical and dry regions. A second intermediate group, with temperatures between 10.0 and 17.5 °C, also follows a logistic curve but with a higher plateau compared to the previous group due to the slower turnover due to lower temperatures. A third group, with temperatures below 10.0 °C, shows an unbounded and broader range of contributions within a narrower range of precipitation, describing circumpolar and alpine regions. Additionally, our results also show an important threshold of around 1,000 mm of annual rainfall where the contribution of TAP becomes positive (above the mean). Areas with precipitation above this threshold are generally located in tropical and temperate regions where water is not a limiting factor for plant development.

### Global additional SOC storage potential

To estimate the carbon stocks, we estimated bulk density using a pedotransfer function generated from the data used in this study (Eq. (3)).

**Figure 5:**
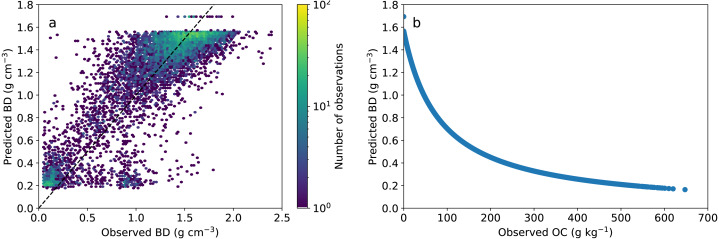
Pedotransfer function performance and relationship with SOC. (A) Observed *vs.* predicted bulk density values. (B) Predicted bulk density showing the decrease in bulk density at higher carbon contents.

(3) \begin{eqnarray*}BD(g~c{m}^{-3})= \frac{83.2687-0.011oc}{0.635oc+52.847} \end{eqnarray*}

where *oc* is the soil organic carbon content in g kg^−1^. This global PTF had a good accuracy, generating an unbiased prediction and a RMSE of 0.25 g cm^−3^. The predicted bulk density also showed the expected behaviour with decreasing values as SOC increases (Fig. 5).

**Figure 6:**
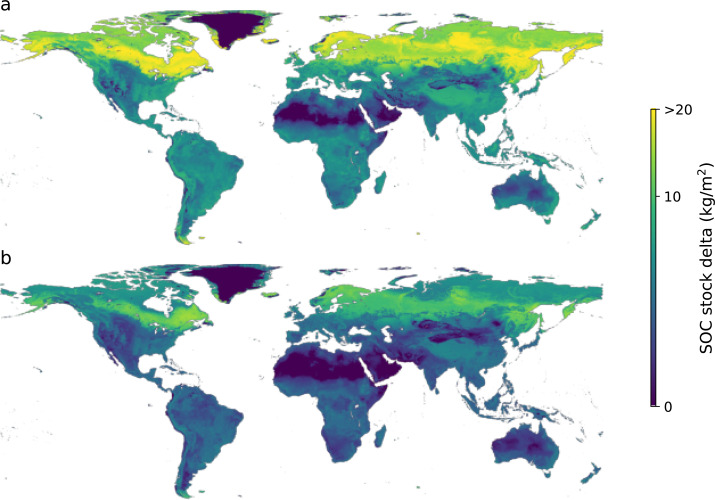
Amount of soil organic carbon required to reach the maximum soil carbon storage capacity to the technical maximum (90th percentile; A) and to the more feasible scenario (75th percentile; B).

Considering the difference between (a) the current and most common practices described by the central tendency of the SOC distribution (50th percentile) and (b) the higher ends of the SOC distribution (75th and 90th percentile) of similar soil under similar climate and defined land cover as the additional storage potential, we estimated its magnitude and spatial distribution at the global scale. Our results show that the soils with the highest SOC additional storage potential are located towards the circumpolar region (Fig. 6), which corresponds to areas with high carbon density (Stockmann et al., 2015). Continental climates present the highest SOC additional storage potential (2.81 kg m^−2^), followed by Temperate and Polar/Alpine regions (both with 1.92 kg m^−2^), Tropical regions (1.59 kg m^−2^) and Dry regions (1.12 kg m^−2^).

Clearly, not all soils can be managed for SOC sequestration, thus we focussed on the topsoil (0–30 cm) of global croplands (1,410 million hectares) which, according to our model (50th percentile), currently contain an estimate of 83 Pg C (Table 1) with a median SOC concentration of 1.37% (or 59.1 Mg C ha^−1^). Our results are lower than the estimate of Zomer et al. (2021) who estimated the global C stock of 131.81 Pg C on 1,593.5 million hectares cropland, or a 82.7 Mg C ha^−1^. A SOC stock of 82.7 Mg C ha^−1^ would be equivalent to 1.9–2.7%, which may be too high for global croplands. Zomer et al. (2021) used the SoilGrids data from Hengl et al. (2017), which could be overestimated (Tifafi, Guenet & Hatté, 2018). Our results show that the areas with the greatest SOC additional storage potential are located in the ecoregions with high pre-cultivation carbon density (*e.g.*, boreal forests/taiga, flooded grasslands and savannas, and temperate broadleaf and mixed forests). Under the practicable scenario, the potential additional topsoil OC storage (the difference between 75th and 50th percentiles) of global cropland is 29 Pg C. Ecoregions of temperate broadleaf and mixed forests, and temperate grasslands, savannas and shrublands account for 59% of the total additional storage potential with 17.2 Pg C. For the technical maximum(difference between 90th and 50th percentiles), the total potential is 65 Pg C, and both ecoregions account for a similar proportion (59%).

**Table 1:** Total topsoil (0–30 cm) SOC stocks (in Pg C) for global croplands by ecoregions.

	Current (q50)	Intermediate (q75)	Technical maximum (q90)	Cropland area (km^2^)
Boreal Forests/Taiga	1.64	2.33	3.06	174,224
Deserts and Xeric Shrublands	3.23	4.45	5.88	1,052,258
Flooded Grasslands and Savannas	0.97	1.41	1.94	145,205
Mangroves	0.20	0.27	0.35	31,880
Mediterranean forests, woodlands and scrub	4.38	5.84	7.58	859,799
Montane grasslands and shrublands	1.29	1.73	2.18	214,602
Temperate broadleaf and mixed forests	29.47	40.38	54.03	4,178,195
Temperate conifer forests	1.15	1.54	2.02	173,098
Temperate grasslands, savannas and shrublands	20.33	26.58	33.91	2,777,674
Tropical and subtropical coniferous forests	0.27	0.37	0.48	43,780
Tropical and subtropical dry broadleaf forests	4.89	6.71	9.25	1,314,051
Tropical and subtropical grasslands, savannas and shrublands	4.92	6.91	9.53	1,262,423
Tropical and subtropical moist broadleaf forests	9.87	13.37	17.48	1,771,950
Tundra	0.05	0.07	0.09	10,712
**Global**	**82.68**	**111.97**	**147.75**	**14,009,852**

**Figure 7:**
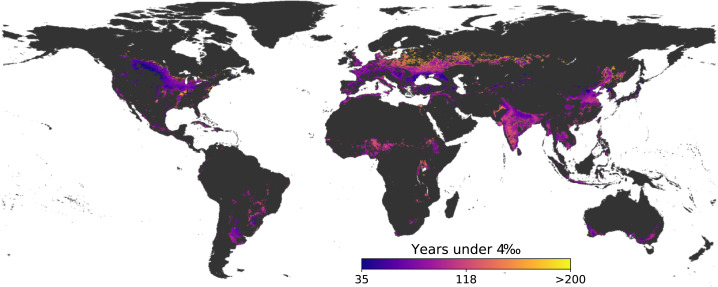
Years to reach the 75th percentile for global croplands. This assumes a fixed 4‰ yr^−1^ accumulation rate which might not be adequate in all cases but it is currently used as an aspirational target.

Recent years have seen increased interest in the potential of improving SOC stock in croplands. For example, the “4 per mille” initiative, launched during COP21 in December 2015 (Minasny et al., 2017), estimates that increasing SOC stocks by 4‰ yr^−1^ could offset some fraction of annual CO_2_ emissions into the atmosphere. There has been a debate on its actual potential as the assumption was made that all soils of the world would increase its SOC more or less uniformly. Accumulation of SOC, regardless of the rate, can only be achieved for a limited time as soils have a natural upper limit for carbon storage which is also limited by management. Using our additional storage potential estimates, we generated global maps simulating a 4‰ yr^−1^ accumulation rate (based on the baseline stock) from the current condition (Fig. 7) and calculated the number of years to reach the practicable and technical maximum. It is important to note that soils will accumulate carbon at different rates, but we used the fixed rate of 4‰ yr^−1^ because it is currently being used to design policies in many places. Our results showed a large spatial variation of the maximum amount of years under the four per mille initiative, with a median period of 86 and 200 years to reach the 75th and 90th percentiles, respectively. For both percentiles, the average capture rate is around 0.34 Pg C yr^−1^ which corresponds to only 4% of the C emissions used to estimate the 4‰ rate (8.9 Pg C yr^−1^) (Minasny et al., 2017).

In addition to using a fixed accumulation rate, the results presented in Fig. 7 assume a linear accumulation. Soils behave differently with SOC accumulation diminishing approximately exponentially in time (Minasny et al., 2017; Franzluebbers et al., 2012) but it might still be a valid estimate since SOC accumulation rates, in many cases, could be greater than 4‰ (Francaviglia et al., 2019). Our estimates are in line with other studies that report croplands reaching a new, higher SOC concentration equilibrium after over a century (Smith, Powlson & Glendining, 1996; Soussana et al., 2004). Regardless of the accumulation rate, the total additional carbon storage potential of the topsoil in croplands is limited. Our total estimates of 29 and 65 Pg C for the 75th and 90th percentiles are equivalent only to three and seven years of global emissions (37.4 Pg CO_2_ eq. yr^−1^ of greenhouse gas derived from anthropogenic activity (Friedlingstein et al., 2022)).

Compared with previous estimates, our results show a slightly higher additional carbon storage potential for global croplands. A total of 18 to 37 Pg C, under medium and high storage scenarios with accumulation rates of 0.9 and 1.85 Pg C yr^−1^ and the assumption of reaching a new equilibrium after 20 years has been reported by Zomer et al. (2017), with estimates based on gridded predictions (Hengl et al., 2017) and considering a uniform sequestration rate for all croplands. A slightly wider range of sequestration potential has been reported by Lal et al. (2018) with a total of 7.63 to 43.25 Pg C over a period of 25 to 50 years, assuming the adoption of region-specific best management practices. According to an extensive review by Fuss et al. (2018), the best estimate of realistic technical potential (close to the median of the minimums of their reviewed studies) is between 20.1 and 46.2 Pg C until 2050. From that year onwards, the accumulation rates could be challenged by sink saturation. It is important to remember that our approach estimates the additional storage potential based on real observations, within a similar climatic context, and not on technical accumulation rates of specific management practices. Since our approach is not based on fixed technical accumulation rates, our results are not necessarily constrained to the 20–50 years period which most studies consider, and could be another reason for our higher estimates.

### Historical carbon debt

Our practicable potential (29 Pg C) is close to the 31.2 Pg C historical debt due to agriculture estimated by a recent study (Sanderman, Hengl & Fiske, 2017). However, we believe that their estimate is too low. In their publication, Sanderman, Hengl & Fiske (2017) report a current SOC stock in global croplands of 62 Pg. If we consider that, in general, soils of agroecosystems contain 25% to 75% less SOC than their counterparts under natural ecosystems (Don, Schumacher & Freibauer, 2011; Lal et al., 2018), the pre-agriculture content would be between 83 and 248 Pg C, which yields a debt between 21 and 186 Pg C, making their estimate very conservative.

Using our model, we estimated a historical carbon debt ranging between 10 and 160 Pg C by replacing all croplands with a range of region-specific natural ecosystems, as described in the methods section. If we consider a midpoint within the latter range as the historical debt due to agriculture (85 Pg C), our practicable potentials account for only 35% of the historical carbon debt (78% for the technical maximum). Here, we only considered croplands as these areas have lost more SOC. There is potential for managed grasslands, however, currently we cannot differentiate between managed and natural grassland using satellite imagery at the global level.

### Effect of climate change

An important point to consider is that carbon the sequestration potential could vary under the future climate. Given the high dependence of SOC on temperature, it is expected that relatively fast global warming will shift most ecosystems toward a lower SOC equilibrium (Figs. 4D–4E) and that this effect will be more pronounced in areas with larger SOC concentrations (Figs. 4A–4C). These projections have been reported in many studies (Robinson, 2007; Crowther et al., 2016; Melillo et al., 2017) and they are likely to result in reduced sequestration potential.

Utilising CMIP6 downscaled future climate projections for a moderate “business as usual” shared socio-economic pathway (SSP3−7.0), we estimated a mean reduction of 14% in the total sequestration potential in croplands in the next 20 years, from 29.9 Pg C to 25.2 Pg C, and from 66.6 Pg C to 56.6 Pg C for the 75th and 90th percentiles, respectively. That estimate does not include the drop in carbon concentration of the current state (50th percentile) which implies an additional loss of 7.9 Pg C.

Note that here we have only included changes that affect our model (*i.e.,* temperature and precipitation) but we have not included the potential changes in net primary productivity (NPP), which is projected to increase in all the CMIP6 scenarios (Gang et al., 2017). Our model does not explicitly consider the relationship between NPP and SOC but it is an implicit factor since there are observations covering a gradient of climatic conditions (and NPP) for a given combination of the rest of the covariates.

### SOC sequestration is still a priority

We have shown that the total amount of additional carbon croplands can store is relatively modest compared to the sustained emission of greenhouse gases derived from anthropogenic activity. It is unreasonable to expect that a single sector can offset global emissions, especially considering their increasing trend. Nevertheless, incorporating carbon into soils by improving management practice should still be a priority to ensure food security. According to our estimates, agriculture generates a carbon debt, so we need to properly manage croplands to be sustainable and avoid the expansion of agricultural land due to the loss of soil productivity.

Agricultural productivity has been directly related to SOC contents. If reduced below some critical limits, soil condition declines and so does crop yield (Lal, 2006; Kopittke et al., 2019). By increasing SOC to its practicable and technical upper limits (75th and 90th percentiles), between 224 and 418 million hectares could be taken above the critical SOC limits of 1.1% and 2% for tropical (Aune & Lal, 1997) and temperate (Loveland & Webb, 2003) areas, respectively.

Several studies have raised concerns about the barriers to sequester SOC (Rumpel et al., 2020). One of the main advantages of our study is that we use a large global database and the estimates are based on real world observations, meaning that a group of locations already reached the target SOC stocks for a given combination of environmental conditions. However, a current limitation to have in mind is that our model is based on biophysical factors but does not take into account socio-economic barriers, disproportionally affecting developing countries, that can impede the adoption of new management practices.

There are still many knowledge gaps that need to be filled and that could help to improve our current model. As mentioned in previous sections, detailed texture information at the global scale is still required and that is also applicable to other soil properties that highly correlate with SOC (Rasmussen et al., 2018). This is necessary to account for local variability. Additionally, our approach places management practices into different quantiles of a distribution based on their SOC density, but it is not capable of identifying them. More research is needed to identify region-specific management practices that can enhance soil carbon. Many countries have a national registry of lands (*e.g.*, Land Parcel Identification in Europe (Leo & Lemoine, 2001)), at least for the management of agricultural subsidies, which should be integrated into soil information systems and added to this type of model. In this work, the selection of such quantiles is illustrative of what we think can be achievable potentials but in practice, any percentile above the median is a gain. The specific percentiles should be adjusted to accommodate different targets, defined by different stakeholders, as concluded by Chen et al. (2019) who applied a similar approach at a national scale in France. In terms of the uncertainty of estimates from this study, they are still large in spite of the methodological improvements. This is the case for all current global carbon stock estimates. Further data collection is required, under different management and biophysical conditions, to increase the confidence of such estimates. Future work should take into account such uncertainty to provide a more robust estimation and potentially guide future soil assessments.

## Conclusions

Our results put in perspective the expectation around soil carbon sequestration in global croplands. They indicate that, even if the additional amount of carbon croplands can accumulate is large (30–67 Pg C), the process is slow, spanning periods up to over a century. Regardless of the accumulation rate, the total additional storage is relatively small compared to the sustained emission of greenhouse gases derived from anthropogenic activity, equating to only three to seven years of emissions offsetting.

Regarding the historical impact of agriculture, our results suggest that the current management practices close to our 75th percentile can only recoup 32% of our estimated 92 Pg C historical debt. Even considering the best current management practices (equivalent to our 90th percentile), we would not be able to recoup that debt fully, only offsetting 72% of them. Hence, agriculture has an intrinsic environmental cost that needs to be taken into account for territorial planning.

Soil carbon capturing measures should be complementary to general carbon emission reduction plans in order to tackle climate change. Moreover, given the high contribution of climate to SOC accumulation, it is expected that the additional SOC storage potential will be affected by climate change if this is not the case. Our results showed that the potential could be reduced by 14% under a moderate “business as usual” shared socio-economic pathway (SSP3-7.0).

The total amount of additional carbon that global croplands can store is relatively small in the context of global carbon emissions. However, it is critical in the context of soil and food security. We estimate that it is possible to restore between 224 and 418 million hectares if we implement management practices conducive to achieve 75th and 90th percentiles. Soil carbon is directly related to agricultural productivity and any improvements in soil condition are a step closer to more sustainable and resilient agricultural systems.

## Additional Information and Declarations

### Data Availability

The following information was supplied regarding data availability: The data is available at WoSIS (Batjes, Ribeiro & Van Oostrum, 2020; doi:10.5194/essd-12-299-2020), CHLSOC (Pfeiffer et al., 2020; doi:10.5194/essd-12-457-2020), and the data described in Stockmann et al. (2015; doi:10.1016/j.gfs.2015.07.001). The land cover information was extracted from the MCD12Q1v6 MODIS product (doi:10.5067/MODIS/MCD12Q1.006).
